# Five-Flavor *Sophora flavescens* Enteric-Coated Capsules for Ulcerative Colitis: A Systematic Review and Meta-Analysis of Randomized Clinical Trials

**DOI:** 10.1155/2022/9633048

**Published:** 2022-01-12

**Authors:** Wen Bin Hou, Wei Jia Sun, Xiao Wen Zhang, Yuan Xi Li, You You Zheng, Yu Xin Sun, Jian Ping Liu, Zhao Lan Liu

**Affiliations:** ^1^Center for Evidence-Based Chinese Medicine, Beijing University of Chinese Medicine, Beijing 100029, China; ^2^Beijing University of Technology, Beijing 100124, China

## Abstract

**Background:**

Ulcerative colitis (UC), a chronic inflammatory bowel disease, is characterized by abdominal pain, diarrhea, and mucopurulent bloody stool. In recent years, the incidence and prevalence of UC have been increasing consistently. Five-flavor *Sophora falvescens* enteric-coated capsule (FSEC), a licensed Chinese patent medicine, was specifically used to treat UC. This review was aimed to assess the effectiveness and safety of FSEC for the treatment of UC.

**Methods:**

Six electronic databases were searched from inception to March 2021. Randomized clinical trials (RCTs) comparing FSEC or FSEC plus conventional Western medicine with conventional Western medicine in participants with UC were included. Two authors screened all references, assessed the risk of bias, and extracted data independently. Binary data were presented as risk ratios (RRs) with 95% confidence intervals (CIs) and metric data as mean difference (MD) with 95% CI. The overall certainty of the evidence was assessed by GRADE.

**Results:**

We included 15 RCTs (1194 participants, 763 in the FSEC group and 431 in the control group). The treatment duration ranged from 42 to 64 days. Twelve trials compared FSEC with conventional Western medicine, and two trials compared FSEC plus conventional medicine with conventional medicine. Another trial compared FSEC plus mesalazine with compound glutamine enteric capsules plus mesalazine. FSEC showed a higher clinical effective rate (improved clinical symptoms, colonoscopy results, and stools) (RR 1.12, 95% CI 1.05 to 1.20; 729 participants; 8 trials; low-quality evidence) as well as the effective rate of traditional Chinese medicine (TCM) syndromes (RR 1.10, 95% CI 1.01 to 1.20; 452 participants; 5 trials; low-quality evidence) compared to mesalazine. There was no significant difference in the adverse events between FSEC and control groups.

**Conclusions:**

FSEC may show effectiveness in UC treatment compared to conventional medicine, and the use of FSEC may not increase the risk of adverse events. Due to the limited number of clinical trials and low methodological quality of the included trials, our findings must be interpreted with discretion.

## 1. Background

Ulcerative colitis (UC), a chronic inflammatory bowel disease (IBD), mainly affects the mucosa and submucosa of the rectum and colon. Its main clinical manifestations include abdominal pain, diarrhea, and mucopurulent bloody stool [1]. UC is characterized by a prolonged disease course and high risk of cancer and is challenging to cure and easy to relapse, which seriously affects the quality of patients' lives [2, 3]. It has been listed as one of the intractable diseases by the World Health Organization and recently became a hot-button issue in digestive diseases. Epidemiological studies showed that the incidence and prevalence of IBD were higher in the Western countries, but the overall trend was stable [4]. In the past 20 years, the incidence and prevalence of IBD in the Eastern countries increased rapidly, and IBD has gradually become a global disease [4–7]. Studies have shown that China is currently one of the countries with the highest incidence of UC in Asia, about 3.44 per 100,000 [8]. With economic development and urbanization, the incidence of UC in China may increase rapidly [9]. Due to the long course and easy recurrence of UC, it brought a severe disease burden to patients. It was estimated that the costs associated with UC are about 12.5 to 29 billion, including 1 billion euros per year in Europe and about 8.1 to 14.9 billion dollars per year in the United States [10]. A German study showed that the average annual treatment cost for patients with UC was 8772.03 euros, and the number of absentee days due to UC was about 16.1 days [11].

At present, the conventional treatment of UC is mainly based on 5-aminosalicylic acid preparations, glucocorticoids, immunosuppressive agents, and biological preparations. However, there are limitations such as poor efficacy for some patients and lower tolerance due to adverse reactions [12–15]. For a long time, traditional Chinese medicine (TCM) has been widely used in the treatment of UC. It has been proved that TCM may control the symptoms of UC patients, accelerate the improvement of the intestinal mucosa, regulate immunity, and improve the quality of life [16–18]. Previously, studies on UC treatment with TCM have been published [19, 20], which found that FSEC is the only Chinese patent medicine licensed for the treatment of UC while the certainty of the evidence is low [21]. The primary raw materials of FSEC are five Chinese medicines such as Sophorae Flavescentis Radix, Sanguisorbae Radix, indigo naturalis, Bletillae Rhizoma, and Glycyrrhizae Radix et Rhizoma, which play a therapeutic role in immune recognition, anti-inflammation, and antioxidation mainly through IL-17, tumor necrosis factor, toll-like receptor, nuclear factor kappa-B, and Th17 cell differentiation [22]. Currently, many randomized clinical trials (RCTs) on FSEC for the treatment of UC are being conducted [23–25]. In light of these published RCTs, we conducted a systematic review and meta-analysis evaluating and verifying the therapeutic effectiveness and safety of FSEC for the treatment of UC.

## 2. Methods

The systematic review protocol was registered in INPLASY (Registration number: INPLASY202150068; available at https://inplasy.com/). We conducted and reported this review according to the Preferred Reporting Items for Systematic Reviews and Meta-Analyses (PRISMA) [26].

### 2.1. Inclusion Criteria

1. Study: we only included RCTs.
2. Participants: patients ≧18 years of age diagnosed with UC, which was defined by clear diagnostic criteria. There was no restriction on patients' gender and course and severity of UC.
3. Interventions: FSEC or FSEC plus conventional medicine, with reporting of the method of medication, dosage, and course of treatment.
4. Controls: conventional Western medicine, with reporting of the method of medication, dosage, and course of treatment.
5. Outcomes: primary outcomes included the clinical effective rate (according to clinical symptoms, colonoscopy inspection, and stool inspection) and colonoscopy curative effect; secondary outcomes covered the disease activity index (DAI), effective rate of traditional Chinese medicine (TCM) syndromes (defined as symptoms and objective signs were improved, with TCM syndrome scores decreased no less than 30%), cytokines, and adverse events.

### 2.2. Study Retrieval and Selection

PubMed, Cochrane Library, Chinese SinoMed, China National Knowledge Infrastructure (CNKI), Chinese Scientific Journal Database (VIP), and Wanfang databases were searched from inception to March 2021. Search terms included mesh terms “Colitis, Ulcerative” and free terms “ulcerative colitis,” “UC,” “composite *Sophora* colon soluble capsules,” “composite *Sophora* enteric-coated capsules,” and “five-flavor *Sophora* enteric-coated capsules.” The retrieval strategies are in Table 1.

WB Hou and JW Sun screened the retrieved articles based on the inclusion criteria by reading their titles, abstracts, and full texts. Any differences were resolved through discussion with a third author (XW Zhang).

### 2.3. Data Extraction and Quality Assessment

WB Hou and XW Zhang extracted the following data into Microsoft Excel 2019 independently and cross checked: basic information of included studies, participants' characteristics, interventions and controls, outcomes, and other relevant information. WB Hou and XW Zhang independently used the Cochrane risk of bias tool [27] to assess the bias of each included trial. Any differences were resolved through discussion with a third author (WJ Sun). Cochrane risk of bias tool consists of the following seven items: random sequence generation, allocation concealment, blinding of participants and personnel, blinding of outcome evaluation, incomplete outcome data, selective reporting, and other biases. Each item was judged as low risk of bias, high risk of bias, or unclear risk of bias.

### 2.4. Data Analysis

We used Review Manager 5.4 software for data analysis. For outcomes, binary data were presented as risk ratio (RR) with 95% confidence interval (CI), and metric data were presented as mean difference (MD) with 95% CI. Statistical analysis was conducted referring to the statistical guidelines from the Cochrane Handbook for Systematic Reviews of Interventions [28]. If the trials showed good homogeneity on study design, participants, interventions, controls, and outcomes, then the meta-analysis would be performed with the random-effects model. We used I^2^ to evaluate statistical heterogeneity. If there were a significant heterogeneity (I^2^ > 90%) between included studies, meta-analysis would not be performed. The source of heterogeneity should be analyzed by subgroup analysis where different types of controls were used. When there were less than 10 RCTs in each meta-analysis, funnel plots would not be used to assess publication bias. In addition, we used the GRADE approach [28] to evaluate the overall certainty of evidence.

## 3. Results

### 3.1. Description of the Literature

**Figure 1:**
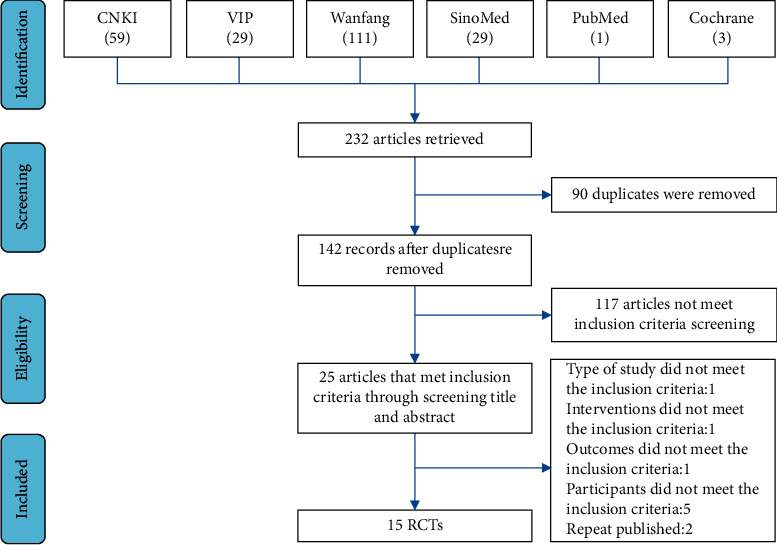
Literature screening and selection flow chart.

A total of 232 articles were retrieved, and 25 remained after screening titles and abstracts. In full-text screening, we excluded ten articles, so 15 RCTs were included in this review finally. The screening process is shown in Figure 1.

### 3.2. Study Characteristics

We included 15 RCTs [23, 25, 29–40] (1194 participants, 763 in the FSEC treatment group and 431 in the control group). Fourteen trials were conducted in China and published in Chinese. Twelve trials compared FSEC versus conventional Western medicine (included mesalazine and SASP), and two compared FSEC plus conventional Western medicine versus conventional Western medicine. Only one trial compared FSEC plus mesalazine versus compound glutamine enteric capsules plus mesalazine. The treatment duration ranged from 42 to 64 days. Participants were aged 18–65 years. The course of illness was 14 days–28 years. There were 501 males and 487 females. Characteristics of included trials are shown in Table 2.

### 3.3. Risk of Bias

**Figure 2:**
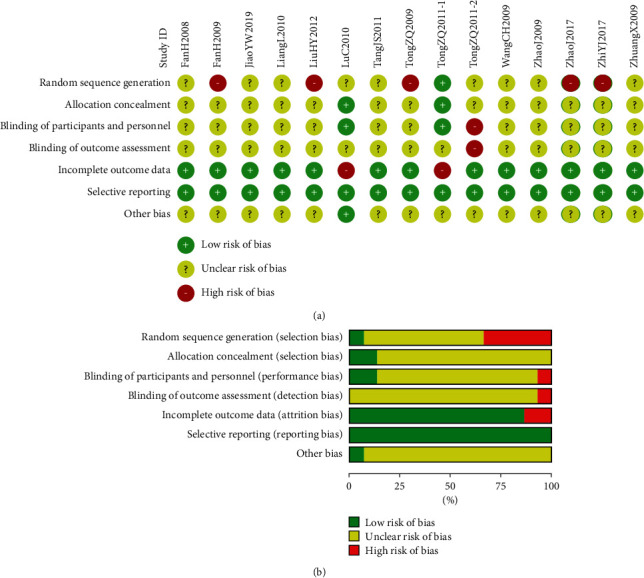
Risk of bias of randomized clinical trials of FSEC for ulcerative colitis. (a) Risk of bias summary. (b) Risk of bias graph.

The risk of bias summary and graph of included trials are given in Figure 2.

### 3.4. Primary Outcomes

#### 3.4.1. Clinical Effective Rate

The clinical effective rate refers to the overall evaluation of the diagnosis and treatment of inflammatory bowel disease [41]. Clinical symptoms and endoscopic examination were used as the evaluation criteria for effectiveness. The clinical effective rate was reported in 14 trials.

**Figure 3:**
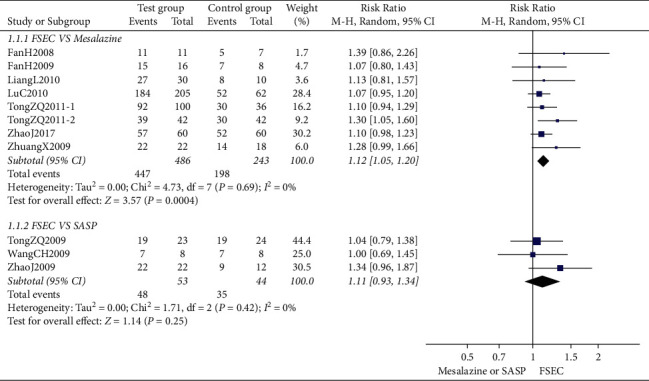
Clinical effective rate of FSEC versus mesalazine and FSEC versus SASP.

*(1) FSEC versus Conventional Treatment*. The clinical effective rate of FSEC alone was 1.12 times more effective than that of mesalazine (RR 1.12, 95% CI 1.05 to 1.20; 729 participants; 8 trials; low-quality evidence) (Figure 3). There was no significant difference between FSEC and SASP on clinical efficacy (RR 1.11, 95% CI 0.93 to 1.34; 97 participants; 3 trials; very-low-quality evidence) (Figure 3).

*(2) FSEC and Conventional Treatment vs. Conventional Treatment*. One RCT compared the clinical efficacy of FSEC combined with mesalazine versus mesalazine, and there was no significant difference (RR 1.17, 95% CI 1.00 to 1.37; 86 participants; 1 trial; low-quality evidence). One trial compared FSEC plus SASP with SASP alone, and the result showed no statistical difference between the two groups (RR 1.29, 95% CI 0.93 to 1.77; 40 participants; 1 trial; low-quality evidence). One trial compared FSEC plus mesalazine with compound glutamine enteric capsules plus mesalazine. The result showed that the FSEC group had worse clinical efficacy than the compound glutamine group (RR 0.79, 95% CI 0.67 to 0.95; 80 participants; 1 trial; low-quality evidence).

#### 3.4.2. Colonoscopy Curative Effect

**Figure 4:**
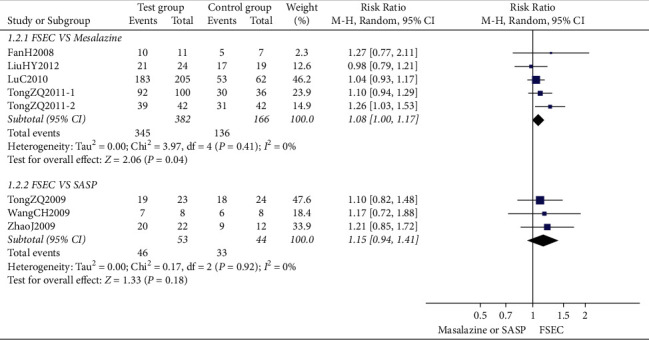
Colonoscopy curative effect of FSEC versus mesalazine.

Eight RCTs reported the colonoscopy curative effect. The meta-analysis showed that there was no significant difference between the colonoscopy curative effect of FSEC and that of mesalazine alone (RR 1.08, 95% CI 1.00 to 1.18; 548 participants; 5 trials; low-quality evidence) (Figure 4). Three trials compared FSEC versus SASP, and the result showed that no significant difference between the two groups (RR 1.15, 95% CI 0.94 to 1.41; 97 participants; 1 trial; low-quality evidence).

### 3.5. Secondary Outcomes

#### 3.5.1. Effective Rate of TCM Syndromes

**Figure 5:**
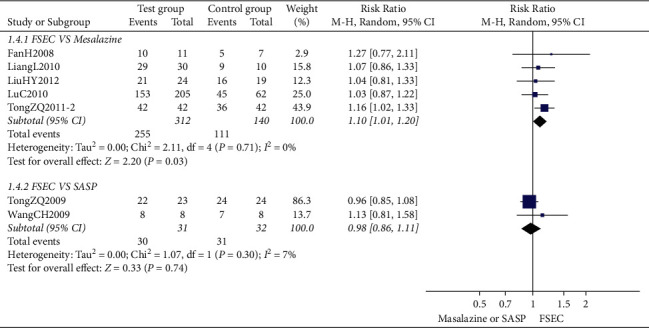
Effective rate of TCM syndromes of FSEC versus mesalazine and FSEC versus SASP.

Seven RCTs reported the effective rate of TCM syndromes. The effective rate of TCM syndromes of FSEC was 1.10 times higher than that of mesalazine alone (RR 1.10, 95% CI 1.01 to 1.20; 452 participants; 5 trials; low-quality evidence) (Figure 5). Two RCTs compared FSEC with SASP, and the result showed that there was no significant difference between the FSEC group and SASP group (RR 0.98, 95% CI 0.86 to 1.11; 63 participants; 2 trials; low-quality evidence) (Figure 5).

#### 3.5.2. Disease Activity Index (DAI)

**Figure 6:**
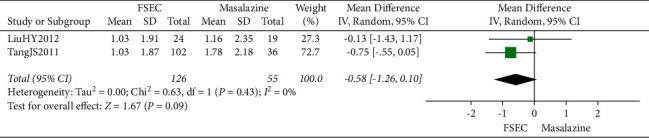
Disease activity index score of FSEC versus mesalazine.

Two RCTs compared FSEC with mesalazine, and the result showed no significant difference between FSEC and mesalazine groups (MD −0.58, 95% CI −1.26 to 0.10; 181 participants; 2 trials; low-quality evidence) (Figure 6).

#### 3.5.3. Cytokine Levels

One trial reported cytokines [23]. The IL-8 level of FSEC combined with the mesalazine group was higher than that of the compound glutamine enteric capsules combined with mesalazine group (MD 0.60 pg/ml, 95% CI 0.05 to 1.15, 80 participants; 1 trial; low-quality evidence). Moreover, the IL-10 level was lower (MD −2.30 ng/ml, 95% CI −4.17 to −0.43, 80 participants; 1 trial; low-quality evidence).

#### 3.5.4. Adverse Events

**Figure 7:**
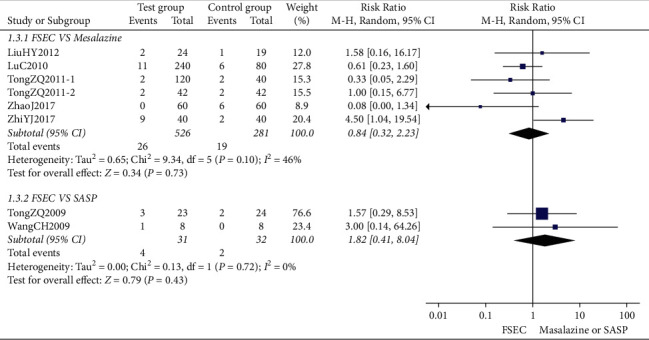
Adverse events of FSEC versus mesalazine and FSEC versus SASP.

Adverse events were reported in 8 out of 15 included RCTs (Table 3). The differences in adverse events between FSEC and control groups are shown in Figure 7.

#### 3.5.5. Publication Bias

There were five types of comparisons in the 15 included trials. Each type of comparison involved no more than ten trials, so inverted funnel plots were not applicable to be conducted to evaluate publication bias.

## 4. Discussion

### 4.1. Summary of Evidence

In this systematic review, the clinical effective rate and effective rate of TCM syndromes of FSEC alone were better than those of mesalazine. However, there was no significant difference in the colonoscopy curative effect between FSEC and mesalazine. In terms of clinical efficacy, the colonoscopy curative effect, the effective rate of TCM syndromes, and DAI, there was no statistical difference between FSEC and SASP or FSEC plus conventional Western medicine and conventional Western medicine. Although some trials had reported adverse events such as nausea, bellyache, and stomachache in the FSEC group, there was also no significant difference in the adverse effects between the experimental group and the control group.

### 4.2. Limitations

The latest consensus added “laboratory examination and imaging examination” to the previous criteria of diagnosing UC, which was based on “clinical manifestations and endoscopic and histopathological manifestations,” emphasizing that the diagnosis of UC needs comprehensive analysis in many aspects [41]. Most of the trials included in this review were too simple in selecting outcome indicators and did not judge the effectiveness by integrating multiple factors.

Sample size estimation is an important measure and premise to ensure the reliability and validity of the study results. A small sample size may lead to false-negative results in the study. Meanwhile, if the sample size were too large, it would increase the difficulty of implementation and waste additional human resources, material resources, and financial resources. The sample size of all trials included was not calculated or estimated in the reports. Most trials enrolled participants of a small sample size which may reduce the credibility of the results.

The formulation and registration of clinical trial protocols can reflect the perspective feature of clinical trials and improve the transparency of clinical trials [18]. However, all trials did not mention the registration of the trial protocol. These studies were short of clinical trial protocols and registration information which may lead to reporting bias and publication bias.

This review systematically collected the evidence from randomized clinical studies whose purpose was to evaluate the effectiveness and safety of FSEC with or without conventional Western medicine on UC. In this review, we conducted a systematic search and strictly assessed the original studies. However, most of the included studies had an unclear risk of bias in terms of random sequence generation, allocation concealment, and blinding. In addition, the quality of evidence included in the studies was generally poor. Although it is undeniable that FSEC may have potential effectiveness in treating UC, more high-quality trials are needed to prove it. Moreover, this review did not limit the searching languages but only retrieved Chinese and English databases, which may also increase the risk of bias. Therefore, we cannot draw firm conclusions based on the evidence of trials included in this review.

According to the results of this study, the outcome indicators included in the study were mostly comprehensive indicators such as the clinical effective rate and effective rate of TCM syndromes. The current research showed that the study of inflammatory cytokines had attracted the attention of a large number of researchers, and inevitable progress had been made in regulating cytokines [42–44]. Therefore, the inclusion of relevant cytokines as outcome indicators may be the direction of improvement of FSEC's future research for the mechanism.

In addition, most of the interventions included in the studies were FSEC alone. Nevertheless, due to the refractory and intractable nature of UC, patients were usually treated with combined therapy of multiple drugs in the clinic [45]. Therefore, more work is needed to bring academic research into clinical implementation and provide practical strategies in the real world.

### 4.3. Comparison with Previous Studies

One related systematic review published in 2018 [21], which included 9 RCTs comparing FSEC alone and Western medicine, found that the total effective rate and improvement of the mucosal lesion had significant differences between groups and no significant difference in the improvement of TCM symptoms and adverse events. In this review, we included all the possible comparisons, including FSEC vs. placebo, FSEC vs. chemical drugs, and FSEC plus chemical drugs vs. chemical drugs. We aimed to provide more comprehensive evidence for clinicians when selecting FSEC as the treatment for UC.

## 5. Conclusions

Based on the evidence in this systematic review, we found that FSEC may have a potentially positive effect on the treatment of UC compared to conventional Western medicine, and the use of FSEC did not increase the risk of adverse events. Due to the limited number of clinical trials and generally poor methodological quality of the included trials, high-quality randomized trials in the future will further validate the effectiveness and safety of FSEC in the treatment of UC.

## Figures and Tables

**Table 1:** Search strategies for each database.

Database name	Search strategies
CNKI	(SU% = “fufang kushen (复方苦参)” OR SU% = “wuwei kushen (五味苦参)” OR SU% = “huibaishi (惠百适)”) AND (SU% = “kuiyangxing jiechangyan (溃疡性结肠炎)” OR SU% = “kuijie (溃结)” OR SU% = “yanzhengxing changbing (炎症性肠病)” OR SU% = “yanxing changbing (炎性肠病)”)
Wanfang	Major Topic (主题): (fufang kushen (复方苦参) OR wuwei kushen (五味苦参) OR huibaishi (惠百适)) AND Major Topic (主题): (kuiyangxing jiechangyan (溃疡性结肠炎) OR kuijie (溃结) OR yanzhengxing changbing (炎症性肠病) OR yanxing changbing (炎性肠病))
VIP	M = (fufang kushen (复方苦参) OR wuwei kushen (五味苦参) OR huibaishi (惠百适)) AND M = (kuiyangxing jiechangyan (溃疡性结肠炎) OR kuijie (溃结) OR yanzhengxing changbing (炎症性肠病) OR yanxing changbing (炎性肠病))
SinoMed	(“Fufang kushen (复方苦参)”[标题:智能] OR “wuwei kushen (五味苦参)”[标题:智能] OR “huibaishi (惠百适)”[标题:智能]) AND (“kuiyangxing jiechangyan (溃疡性结肠炎)”[标题:智能] OR “Kuijie (溃结)”[标题:智能] OR “yanzhengxing changbing (炎症性肠病)”[标题:智能] OR “yanxing changbing (炎性肠病)”[标题:智能])
PubMed	#1 Search: (composite *Sophora* colon soluble capsules [title/abstract]) OR (composite *Sophora* enteric-coated capsules [title/abstract]) OR (five-flavor *Sophora* enteric-coated capsules [title/abstract])
	#2 Search: ((colitis, ulcerative [MeSH terms]) OR (ulcerative colitis [title/abstract])) OR (UC [title/abstract])
	#3 Search: (#1) AND (#2)

Cochrane Library	#1 Search: MeSH descriptor:[colitis, ulcerative] this term only
	#2 Search: (ulcerative colitis):ti,ab,kw OR (UC):ti,ab,kw
	#3 Search: (composite *Sophora* colon soluble capsules):ti,ab,kw OR (composite *Sophora* enteric-coated capsules):ti,ab,kw OR (five-flavor *Sophora* enteric-coated capsules):ti,ab,kw
	#4 Search: #1 OR #2 AND #3

**Table 2:** Characteristics of included randomized trials of FSEC for ulcerative colitis.

Study ID	Year	Gender (male/female)	Sample size	Age (mean or range, years)	Course of disease (mean or range)	Duration of treatment (days)	Intervention vs. control	Intervention details	Controls	Outcomes
ZhiYJ2017	2017	42/38	I:40	I:38.8 ± 3.9	I: 6 months–8 years	42	FSEC + mesalazine vs. compound glutamine enteric capsules + mesalazine	FSEC: 3 times/day, 1.6 g/time	Compound glutamine enteric capsules: 3 times/day, 3 pills/time	(1), (5), (6), (7)
			C:40	C:39.4 ± 4.2	C: 2 months–9 years			Mesalazine: 4 times/day, 1 g/time	Mesalazine: 4 times/day, 1 g/time	

JiaoYW2019	2019	I:21/22	I:43	I:57 ± 12	I: 6.8 ± 2.3 years	56	FSEC + mesalazine vs. mesalazine	FSEC: 3 times/day, 1.6 g/time	Mesalazine: 4 times/day, 1 g/time	(1)
		C:20/23	C:43	C:59 ± 12	C: 7.2 ± 2.5 years			Mesalazine: 4 times/day, 1 g/time		

ZhaoJ2017	2017	74/46	I:60	20–60 (42.56 ± 3.26)	1–15 (7.01 ± 1.24) years	64	FSEC vs. mesalazine	FSEC: 3 times/day, 1.6 g/time	Mesalazine: 4 times/day, 1 g/time	(1), (5)
			C:60							

LiuHY2012	2012	I:8/16	I:24	26–64 (48.14 ± 8.72)	19–62 (46.49 ± 10.02) years	56	FSEC + mesalazine placebo vs. mesalazine + FSEC placebo	FSEC: 3 times/day, 1.6 g/time;	Mesalazine: 4 times/day, 1 g/time	(2), (3), (4), (5)
		C:6/13	C:19					Mesalazine placebo: 4 times/day, 1 g/time	FSEC placebo: 3 times/day, 1.6 g/time	

TangJS2011	2011	17/23	I: 20	34 years on average	6 months–10 years	56	FSEC + SASP vs. SASP	FSEC: 3 times/day, 1.6 g/time;	SASP: 4 times/day, 1 g/time	(1)
			C: 20					SASP: 4 times/day, 1 g/time		

TongZQ2011-1	2011	I: 59/61	I: 120	I: 42.88 ± 11.77	NR	56	FSEC + mesalazine placebo vs. mesalazine + FSEC placebo	FSEC: 3 times/day, 1.6 g/time;	Mesalazine:4 times/day, 1 g/time	(1), (2), (4), (5)
		C: 23/17	C: 40	C: 42.70 ± 10.42				Mesalazine placebo: 4 times/day, 1 g/time	FSEC placebo: 3 times/day, 1.6 g/time	

LiangL2010	2010	I: 18/12	I: 30	I: 18–65 (43.28 ± 12.33)	14 days–20 years	56	FSEC + mesalazine placebo vs. mesalazine + FSEC placebo	FSEC: 3 times/day, 1.6 g/time;	Mesalazine:4 times/day, 1 g/time	(1), (3)
		C: 6/4	C: 10	C: 18–65 (42.78 ± 12.36)				Mesalazine placebo: 4 times/day, 1 g/time	FSEC placebo: 3 times/day, 1.6 g/time	

TongZQ2011-2	2011	I: 20/22	I: 42	I: 38.7 ± 10.8	NR	56	FSEC vs. mesalazine	FSEC: 3 times/day, 2.4 g/time	Mesalazine:4 times/day, 1 g/time	(1), (2), (3), (5)
		C: 21/21	C: 42	C: 41.2 ± 12.3						
		I: 24/18	I: 42	I: 40.7 ± 10.2	NR	56	FSEC vs. mesalazine	FSEC: 3 times/day, 1.6 g/time	Mesalazine:4 times/day, 1 g/time	
		C: 21/21	C: 42	C: 41.2 ± 12.3						

LuC2010	2010	I: 126/114	I: 240	I: 43.63 ± 12.02	NR	56	FSEC + mesalazine placebo vs. mesalazine + FSEC placebo	FSEC: 3 times/day, 1.6 g/time;	Mesalazine:4 times/day, 1 g/time	(1), (2), (3), (5)
		C: 38/42	C: 80	C: 44.51 ± 12.02				Mesalazine placebo: 4 times/day, 1 g/time	FSEC placebo: 3 times/day, 1.6 g/time	

TongZQ2009	2009	I: 10/13	I: 23	I: 44.39 ± 12.99	NR	56	FSEC vs. SASP	FSEC: 3 times/day, 1.6 g/time	SASP: 4 times/day, 0.75 g/time	(1), (2) ,(3), (5)
		C: 13/11	C: 24	C: 38.25 ± 13.19						

WangCH2009	2009	I: NR	I: 8	18–65	NR	56	FSEC vs. SASP	FSEC: 3 times/day, 1.6 g/time	SASP: 4 times/day, 0.75 g/time	(1), (2), (3), (5)
		C: NR	C: 8							

FanH2009	2009	I: NR	I: 16	18–65	NR	56	FSEC vs. mesalazine	FSEC: 3 times/day, 1.6 g/time	Mesalazine: 4 times/day, 1 g/time	(1)
		C: NR	C: 8							

ZhaoJ2009	2009	I: 5/17	I: 22	I: 25–64 (32.51 ± 12.46)	I: 1.5–25 years	56	FSEC vs. SASP	FSEC: 3 times/day, 1.6 g/time	SASP: 4 times/day, 1 g/time	(1), (2)
		C: 6/6	C: 12	C: 24–62 (31.73 ± 11.58)	C: 2–28 years					

ZhuangX2009	2009	I: NR	I: 22	NR	NR	56	FSEC vs. mesalazine	FSEC: 3 times/day, 1.6 g/time	Mesalazine: 4 times/day, 1 g/time	(1)
		C: NR	C: 18							

FanH2008	2008	I: 6/5	I: 11	I: 25–59	I: 1.5–25 years	56	FSEC vs. mesalazine	FSEC: 3 times/day, 1.6 g/time	Mesalazine: 4 times/day, 1 g/time	(1), (2), (3)
		C: 3/4	C: 7	C: 24–58	C: 1.5–28 years					

I, intervention group; C, control group; FSEC, five-flavor *Sophora flavescens* enteric-coated capsule; SASP, sulfasalazine; DAI, disease activity index; TCM, traditional Chinese medicine; NR, not reported. (1) Clinical effective rate, (2) colonoscopy curative effect, (3) effective rate of TCM syndromes, (4) DAI, (5) adverse effects, (6) IL-8, (7) IL-10.

**Table 3:** Adverse events in included studies.

Study ID	Number of adverse events	Intervention	Control
ZhiYJ2017	I: 9/40	5 nausea, 2 stomach discomfort, and 2 other adverse reactions	1 nausea and 1 stomach discomfort
	C: 2/40		

ZhaoJ2017	I: 0/60	—	4 nausea and 2 pruritus
	C: 6/60		

LiuHY2012	I: 2/24	1 pharyngitis and 1 bellyache	1 fever
	C: 1/19		

TongZQ2011-1	I: 2/120	1 indigestion and 1 menstrual disorder	1 insomnia and 1 fatigue
	C: 2/40		

TongZQ2011-2	I: 2/42	1 pharyngitis and 1 nausea	1 fatigue and 1 general aching
	C: 2/42		

LuC2010	I: 11/240	1 nausea, 2 fatigue, 2 bellyache, 3 abdominal distension, 1 hepatic discomfort, 1 perianal pain, and 1 decreased appetite	3 bellyache, 1 abdominal distension, 1 upper respiratory infection, and 1 fever
	C: 6/80		

TongZQ2009	I: 3/23	1 bellyache, 1 stomachache, and 1 stomach discomfort	1 oral ulcer and 1 painful pharynx
	C: 2/24		

WangCH2009	I: 1/8	1 bellyache	—
	C: 0/8		

## Data Availability

All data analyzed in this study are supported by the published articles in databases, including six opening electronic databases (details in study identification and selection). All data generated are included in this published article.

## Abbreviations

RCTs: Randomized clinical trials
UC: Ulcerative colitis
CNKI: China National Knowledge Infrastructure
VIP: Chinese Scientific Journal Database
CI: Confidence interval
MD: Mean difference
RR: Relative risk
I: Intervention
C: Control
NR: Not reported
Y: Yes
N: No.

## Supplementary Materials

Supplementary Materials PRISMA 2020 checklist.
